# Neonatal overfeeding induced glucocorticoid overexposure accelerates hepatic lipogenesis in male rats

**DOI:** 10.1186/s12986-018-0272-0

**Published:** 2018-05-02

**Authors:** Fan Yang, Yanyan Dai, Cuiting Min, Xiaonan Li

**Affiliations:** 1 Department of Child Health Care, Children’s Hospital of Nanjing Medical University, 72 Guangzhou Road, Nanjing, China; 2 Institute of Paediatric Research, Nanjing Medical University, 140 Hanzhong Road, Nanjing, China

**Keywords:** Glucocorticoid, NAFLD, Postnatal overfeeding, 11β-HSD1, Glucocorticoid receptor

## Abstract

**Background:**

Postnatal overfeeding activates tissue glucocorticoid (GC) activity by up-regulating 11β-hydroxysteroid dehydrogenase 1 (11β-HSD1) and increasing sensitivity to high-fat (HF) diet-induced non-alcoholic fatty liver disease (NAFLD). The present study aimed to evaluate the effects of postnatal overfeeding on GC regulation and lipogenesis in the liver and to observe the impact of GC on hepatocyte lipid metabolism.

**Methods:**

In vivo*,* Male Sprague-Dawley rat pup litters were adjusted to litter sizes of three (small litter, SL) or ten (normal litter, NL) on postnatal day 3 and then given standard chow from postnatal week 3 (W3) to W13. In vitro*,* HepG2 cells were stimulated by GC, mifepristone (Mi) or GC + Mi within 48 h, followed by sodium oleate (OA) intervention (or not) for 24 h. Intracellular lipid droplets, triglyceride (TG) concentrations and gene expression related to lipid metabolism were measured in hepatic tissues or HepG2 cells.

**Results:**

In vivo*,* weight gain in the body and liver and TG concentrations in the liver were significantly increased in the SL rats compared to the NL rats at W3 and W13 (*p* < 0.05); mRNA expression of hepatic 11β-HSD1, acetyl-CoA carboxylase 1 (ACC), stearoyl-CoA desaturase-1 (SCD1), fatty acid synthase (FASN) and their nuclear transcription factor, sterol regulatory element binding protein-1c (SREBP-1c) (*p* < 0.05), was also increased. In vitro, intracellular lipid droplets and TG content in HepG2 cells increased under stimulation with GC or OA (*p* < 0.05); the increase was more significant following treatment with GC and OA together (*p* < 0.05). The ACC, SCD1, FASN and SREBP-1c mRNA expression changes were highly similar to the changes in TG content in cells. All the changes induced by GC disappeared when the glucocorticoid receptor (GR) was blocked by Mi.

**Conclusions:**

Postnatal overfeeding induced GC overexposure through 11β-HSD1 up-regulation in the liver. GC activated hepatic de novo lipogenesis (DNL) via GR and led to hepatic lipid accumulation, which increased the risk of NAFLD during adulthood.

## Background

Non-alcoholic fatty liver disease (NAFLD) and other components of metabolic syndrome (MS) have become increasingly comorbid with the increasing prevalence of obesity in both children and adults [1, 2]. Unlike what happens in adults, the onset of paediatric NAFLD is asymptomatic until it progresses into hepatic fibrosis and cirrhosis [3, 4]. In order to develop strategies to effectively prevent NAFLD and MS, it is important to better understand the mechanisms by which obesity increases susceptibility to NAFLD.

NAFLD is characterized by excessive triglyceride (TG) accumulation in the absence of significant alcohol consumption [1]. It is primarily caused by the imbalance of hepatic lipid homeostasis between the acquisition and removal of TG/fatty acid, which involves increased fatty acid/TG uptake, enhanced de novo lipogenesis (DNL), impaired fatty acid β-oxidation, and/or decreased lipid export in the liver. Several rate-limiting enzymes and transcription factors participate in hepatic lipid metabolism [5]. Hepatic lipoprotein lipase (LPL) and liver-type fatty acid-binding protein (L-FABP) are thought to play a central role in hepatic lipid uptake [6, 7]. The activation of DNL to TG synthesis in the liver involves sterol regulatory element binding protein-1c (SREBP-1c) and lipogenic enzymes including fatty acid synthase (FASN), acetyl-CoA carboxylase 1 (ACC) and stearoyl-CoA desaturase-1 (SCD1) [8, 9]. In addition, fatty acid β-oxidation and lipid export in the liver involve peroxisome proliferator-activated receptor α (PPARα), carnitine palmitoyltransferase 1 (CPT1) and microsomal triglyceride transfer protein (MTP) [10–15]. The activities of these enzyme systems are regulated by nutrition, the endocrine system and inflammation [16–18].

Glucocorticoid (GC), such as corticosterone and cortisol, affects fat accumulation and lipid and glucose metabolism [19, 20]. At the tissue level, GC exposure is determined not only by circulating levels, but also by the tissue-specific GC-activating enzyme 11β-hydroxysteroid dehydrogenase type 1 (11β-HSD1) and the GC-inactivating enzymes 5α-reductase type 1 (5αR1) and 5β-reductase (5βR) [21, 22]. Previous studies have shown that the overexpression of 11β-HSD1 in tissue amplifies local GC action, which leads to increased accumulation of adipose tissue and metabolic disorders in both humans and rodents [23–26]. GC is involved in every stage of the pathogenesis of NAFLD [27]. In animal models, GC increases lipid biosynthesis within the liver that can lead to hepatic steatosis and increase circulating TG levels [28, 29].

The results of both experimental studies using animal models and clinical investigations have indicated that the early nutrition environment is associated with the development of obesity and MS in later life [30–33] and that GC is a possible mediator of the permanent programming of obesity, insulin resistance, and other metabolic dysregulations [34–36]. Previously, we reported that small litter (SL) rearing induced obesity in adult rats. The animals also had hyperinsulinemia, elevated circulating corticosterone levels, peripheral tissue-specific alterations in 11β-HSD1 expression and activity and 5αR1 and 5βR expression starting at puberty [37]. In addition, SL rats also displayed increased ACC activation in the livers and were more prone to develop NAFLD when challenged with high-fat (HF) diets [38]. Our hypothesis was that local GC activity plays the crucial role in the pathogenesis of hepatic steatosis by regulating lipid synthesis enzymes. Therefore, in this study we examined the expression patterns of 11β-HSD1 and 5αR1 and 5βR in the livers of SL rats, as well as those of lipid metabolism-related genes involved in hepatic DNL and fatty acid β-oxidation and lipid export. Moreover, we wanted to determine the action of GC on hepatic lipid metabolism. We first treated HepG2 cells with GC and then with OA in vitro to mimic hepatic GC overexposure in vivo*.*

## Methods

### Animals and experimental design

All animal studies were performed following the guidelines established by the University Committee on the Use and Care of Animals and were overseen by the Unit for Laboratory Animal Medicine at Nanjing Medical University (IACUC: 14030102). Male Sprague-Dawley rats were used. They were maintained under a controlled 12/12 h light/dark cycle in temperature (22 ± 2 °C) conditions with free access to food and water.

The experimental setup was similar to that described in Boullu-Ciocca [39]. In rats, the weaning period is postnatal week 3, puberty is postnatal weeks 6-8 and adulthood is week 9 and afterward [40]. In our previous studies, we showed that metabolism disorders in SL rats took place during postnatal weeks 13-16 [38, 41]. Therefore, postnatal weeks 3 and 13 were selected as two experimental points of this study to examine the effects of early nutrition on adult health. On postnatal day 3 (P3), male pups were randomly redistributed to litter sizes of three (SLs) or ten (normal litters (NLs)) to induce early postnatal overfeeding or normal nutrition, respectively [42]. After weaning (P21, W3), the NL and SL rats were fed a standard diet (NL or SL group) until postnatal week 13 (W13). All rats were housed 3-4 per cage after weaning. Body weight and food intake were monitored weekly throughout life. The animals were killed at W3 and W13 after an overnight fast.

### Tissue collection

The rats were anaesthetized with chloral hydrate (300 mg/kg body weight, i.p.) after an overnight fast (12 h). Body weight was recorded. Each rat’s liver was dissected and weighed, and the hepatosomatic index (HSI) was calculated as (liver weight/body weight) * 100% [43]. All tissue samples were snap-frozen in liquid nitrogen and stored at − 70 °C until gene expression analysis.

### Hepatic lipid assays

Concentrations of TG in the liver and cells were determined using TG assay kits (E1013, Applygen, Beijing, China). The hepatic TG concentration was expressed relative to 1 g of liver protein. Hepatic protein concentrations were determined using a Pierce BCA protein assay kit with bovine serum albumin as the standard (Thermo Fisher Scientific, Rockford, IL, USA).

### Cell culture

HepG2 cells, obtained from Keygen Biotech (Nanjing, China, ATCC HB-8065), were maintained in DMEM medium containing 10% FBS and 1% P/S at 37 °C with 5% CO_2_ (Thermo Scientific, CO_2_ incubator) in 75 cm^2^ flasks. Cells were plated in 6-well plates at 2*10^5^ cells per well. The following day, confluent cells were starved for 6 h without FBS. Then, the cells were treated with 2.0 ml of fresh supplemented culture medium containing dexamethasone (active GC, D4902, Sigma), mifepristone (glucocorticoid receptor (GR) antagonist, Mi, M8046, Sigma), both GC and Mi, or vehicle (culture medium) for 48 h, followed by exposure (or not) to sodium oleate (OA, O7501, Sigma), which is rich in fatty acids, for 24 h. To evaluate the possible effects of GC on gene expression related to lipid metabolism, HepG2 cells were incubated with GC at different concentrations (0, 50, 100, 125, 250, 500 and 1000 nM; *n* = 3 for each concentration) and time (24, 36 and 48 h) and to ascertain the maximal response. The effects of GC (125 nM) combined with Mi at different concentrations (0, 0.1, 1, 5, 10 μM) were then used (*n* = 3) to evaluate the individual and combined effects on the hepatic lipid homeostasis. The TG content in the cells was determined using commercial kits (E1013, Applygen, Beijing, China).

### Oil red O staining

At the end of incubation, the cultured cells were washed with PBS and fixed with 4% formaldehyde for 30 min at room temperature. Then, the cells were stained using Oil red O working solutions containing 6 ml of Oil red O stock solution (0.5 g in 100 ml of isopropanol) and 4 ml of ddH_2_O at 37 °C for 30 min. Staining was visualized by bright-field microscopy (BX51, OLYMPUS, Japan).

### Total RNA extraction and real-time PCR

Total RNA was extracted from cells or liver tissues using Trizol (Invitrogen) according to the manufacturer’s instructions and quantified spectrophotometrically at OD260. The integrity of the total RNA was assessed using agarose gel electrophoresis, and cDNA was synthesized using M-MLV reverse transcriptase (TAKARA) with 0.5 μg of the RNA sample as recommended by the manufacturer. Genes of interest were analysed by real-time PCR using the SYBR GREEN ABI Prism 7500 sequence detector for the target genes, including SREBP-1c, ACC, SCD1, FASN, PPARα, LPL, L-FABP, CPT1 and MTP (Table 1). Expression of the target genes was normalized to the expression of glyceraldehyde-3-phosphate dehydrogenase (GAPDH) (Table 1).

**Table 1:** Primer sequences used for mRNA quantification by real-time PCR

	Forward primer 5′–3′	Forward primer 5′–3′
ACC rat	TGAAGGGCTACCTCTAATG	TCACAACCCAAGAACCAC
SCD1 rat	CTCCCTACCTCCACCCCTAT	AACCAACCCTCTCGTTCAGT
FASN rat	AAGAGTGGGAGAGCCTGTTC	AGTTCACCAAGCCTACCACA
LPL rat	GCTTCCCCTTACTGGTTCC	AACTGGCAGGCAATGAGACT
L-FABP rat	AAGGGAAGGACATCAAGGGG	CACTGCCTTGACCTTTTCCC
CPT1 rat	ACGAAGAACATTGTGAGCGG	GAGGACCTTGACCATAGCCA
MTP rat	AGCAACATGCCTACTTCTTACAC	TCACGGGTTCACTTTCACTG
SREBP-1c rat	CGCTACCGTTCCTCTATCA	CTCCTCCACTGCCACAAG
PPARα rat	AGCCATTCTGCGACATCA	CGTCTGACTCGGTCTTCTTG
GAPDH rat	GGCTCTCTGCTCCTCCCTGTTCTA	CGTCCGATACGGCCAAATCCGT
ACC hum	CACGCTCAAGTCACCAAGAA	GCAAATGGGAGGCAATAAGA
SCD1 hum	GTTCGTTGCCACTTTCTTGC	TGGTAGTTGTGGAAGCCCTC
FASN hum	CGGTGTTTGAGTTCGTGGAG	CGGGGATAGAGGTGCTGAG
LPL hum	TTCTCGTTGGCAGGGTTGAT	CTGACACTGTTTTCACGCCA
L-FABP hum	TTCAAGTTCACCATCACCGC	TTATGTCGCCGTTGAGTTCG
CPT1 hum	CAACTCACATTCAGGCAGCA	CGATGTGCTTGCTGTCTCTC
MTP hum	CTGCTCAGACCTCAGACTCA	TCTCTGATGTCACTGCTACCA
SREBP-1c hum	TTCCCAGCCCCTCAGATAC	GAGAAGCACCAAGGAGACGA
PPARα hum	CCAGCATCCTCTCTCCAACT	AGGAAAACGAAGACCCAAGAT
GAPDH hum	GTCGGAGTCAACGGATTTGG	CATGGGTGGAATCATATTGGA

### Statistical methods

Data are expressed as Means ± SEM. Two-ways analysis of variance (ANOVA) tests were used to analyse body weight gain. Two-sided Student’s *t*-test was used to analyse liver weight, hepatic lipid content, mRNA expression and the effects of different treatments in cell culture. Statistical significance was accepted at *p* < 0.05.

## Results

### Food intake and body weight

**Fig. 1:**
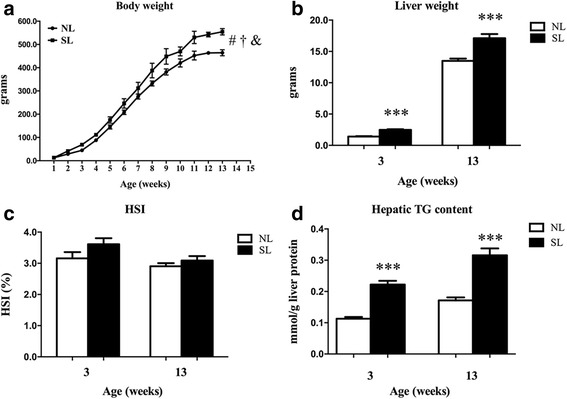
Body weight (**a**) from postnatal week 1 to week 13; liver weight (**b**), hepatosomatic index (**c**) and hepatic TG content (**d**) in normal litters (NLs) and small litters (SLs) at week 3 (W3) and week 13 (W13). Data are expressed as the mean ± SEM. Body weight gain was analyzed by two-way ANOVA. ^#^*F* = 1980, *p* < 0.001 for effect of age; ^†^*F* = 363, *p* < 0.001 for effect of SL; ^&^*F* = 11, *p* < 0.001 for interaction of SL and age. Significant differences between groups at W3 or W13 were analyzed by two-sided Student’s *t*-test. ***p < 0.001 vs. NL rats. *n* = 6 in each NL and SL group

The food intake of the SL rats increased significantly only at W3 to W5 compared to the NL rats (*p* < 0.01, Table 2), and there were no significant differences between groups after that time (*p* > 0.05, Table 2). Body weight increased with age in both groups (*p* < 0.001), and the SL rats gained more than the NL rats (*p* < 0.001); there was a significant interaction for weight gain in the SL rats with age (*p* < 0.001, Fig. 1a).

**Table 2:** Food intake at W3, 4, 5, 6, 8, and 13 in NL and SL rats

	NL	SL
Food intake (g/day)		
3 wk	9.1 ± 0.2	14.3 ± 0.5***
4 wk	10.6 ± 0.1	18.9 ± 0.3***
5 wk	14.1 ± 0.7	18.0 ± 0.5**
6 wk	22.1 ± 0.2	22.0 ± 0.3
8 wk	26.6 ± 0.2	26.4 ± 0.1
13 wk	27.5 ± 0.6	27.8 ± 0.2

Data are expressed as the mean ± SEM. Significant differences between groups of rats at corresponding time points were analyzed by two-sided Student’s *t*-test

**p < 0.01, ***p < 0.001 vs. NL rats. *n* = 6 in each NL and SL group

### Liver weight and hepatic TG content

The liver weight was higher in the SL rats compared to the NL rats at W3 and W13 (*p* < 0.001, Fig. 1b), but there was no significant difference in HSI between groups (*p* > 0.05, Fig. 1c). Hepatic TG content was higher in the SL rats compared to the NL rats at W3 and W13 (*p* < 0.001, Fig. 1d).

### 11β-HSD1, 5αR1 and 5βR mRNA expression in the liver at W3 and W13

**Fig. 2:**
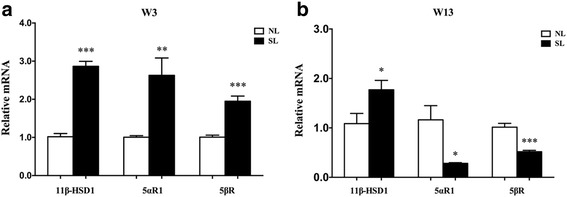
mRNA expression of 11β-HSD1, 5αR1 and 5βR at W3 (**a**) and W13 (**b**). Data are expressed as the mean ± SEM. Significant differences between groups at W3 or W13 were analyzed by two-sided Student’s *t*-test. *p < 0.05, **p < 0.01, ***p < 0.001 vs. NL rats. *n* = 6 in each NL and SL group

Hepatic 11β-HSD1 mRNA expression was higher in the SL rats compared to the NL rats at W3 (*p* < 0.001, Fig. 2a) and W13 (*p* < 0.05, Fig. 2b). Compared to the NL rats, hepatic 5αR1 and 5βR mRNA expression was higher in the SL rats at W3 (*p* < 0.01, Fig. 2a) but decreased significantly in the SL rats compared to the NL rats at W13 (*p* < 0.01, Fig. 2b).

### mRNA expression of rate-limiting enzymes in hepatic tissue at W3 and W13

**Fig. 3:**
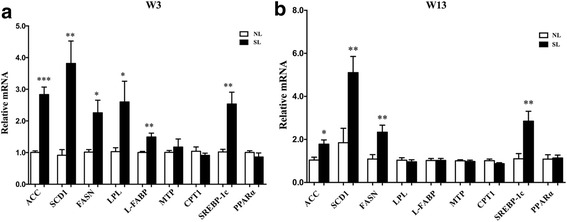
mRNA expression of the genes involved in hepatic lipid metabolism at W3 (**a**) and W13 (**b**). Data are expressed as the mean ± SEM. Significant differences between groups at W3 or W13 were analyzed by two-sided Student’s *t*-test. *p < 0.05, **p < 0.01, ***p < 0.001 vs. NL rats. *n* = 6 in each NL and SL group

Hepatic ACC, SCD1, FASN and SREBP-1c mRNA expression was significantly increased in the SL rats compared to the NL rats at W3 and W13 (*p* < 0.05, Fig. 3), whereas mRNA expression of LPL and L-FABP mRNA only increased in the SL rats at W3 (*p* < 0.05, Fig. 3a), but not at W13 (*p* > 0.05, Fig. 3b). There were no significant differences in the expression of PPARα, CPT1 or MTP between the two groups at W3 or W13 (*p* > 0.05, Fig. 3).

### Effects of GC or/and OA on lipid accumulation in HepG2 cells

**Fig. 4:**
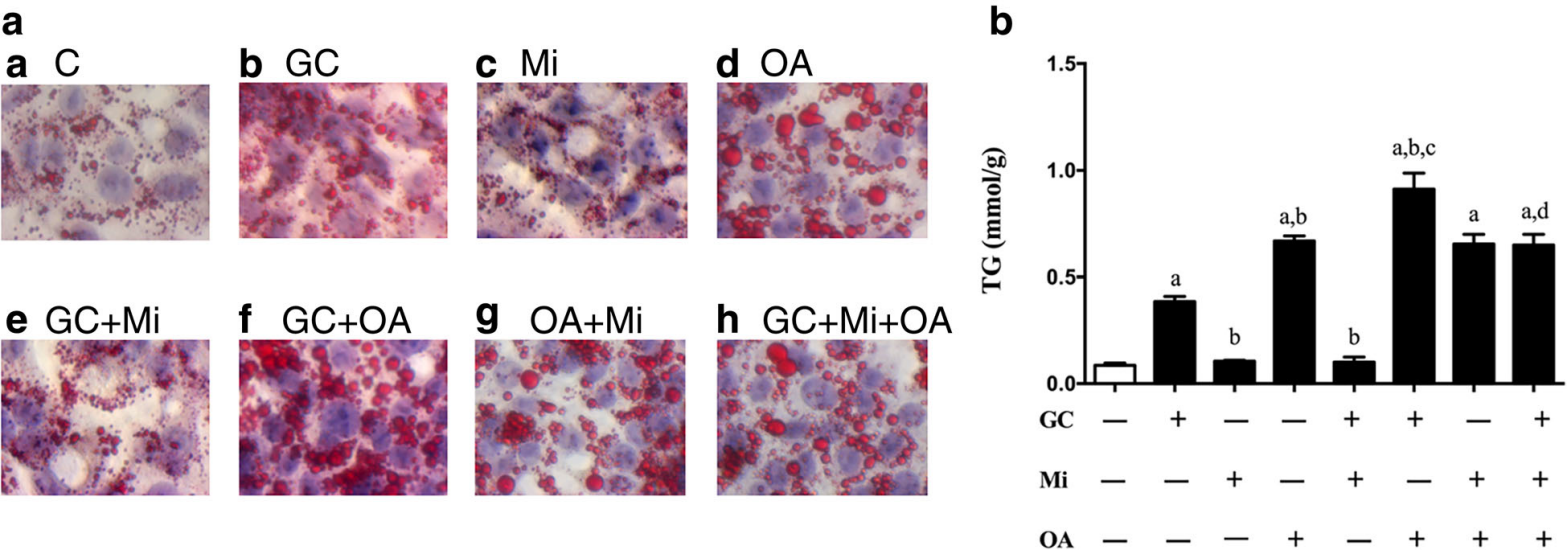
Oil red O staining of cells after treatment with GC, Mi, or GC + Mi within the first 48 h, followed by sodium oleate (OA) intervention (or not) for 24 h (**a**). The TG contents are shown in (**b**). Data are expressed as the mean ± SEM. Effects of different treatments were analyzed by two-sided Student’s *t*-test. ^a^*p* < 0.05 vs. Control, ^b^*p* < 0.05 vs. GC; ^c^*p* < 0.05 vs. OA; ^d^*p* < 0.05 vs. GC + OA. *n* = 3 in each treatment

Oil red O staining showed that little lipid droplets existed in the normal HepG2 cells, but these intracellular lipid droplets were obviously increased in the cells after treatment with GC or OA; there was more significant lipid accumulation after treatment with GC + OA (Fig. 4a). In addition, Mi treatment attenuated the increase of the lipid accumulation induced by GC or GC + OA (Fig. 4a). Like the lipid droplet accumulation, the TG content in the HepG2 cells increased after treatment with GC or OA or GC + OA and decreased when Mi was added compared to the GC or GC + OA treatment (*p* < 0.05, Fig. 4b).

### Gene expression in response to GC and OA in HepG2 cells

**Fig. 5:**
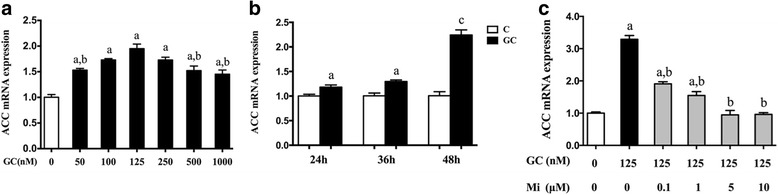
ACC mRNA expression was dependent on the GC treatment concentrations (**a**), timing (**b**) and treatment with GC (125 nM) and Mi at different concentrations (**c**). Data are expressed as the mean ± SEM. Effects of different treatments were analyzed by two-sided Student’s *t*-test. ^a^*p* < 0.05 vs. control, ^b^*p* < 0.05 vs. GC (125 nM), ^c^*p* < 0.01 vs. control. *n* = 3 in each treatment

**Fig. 6:**
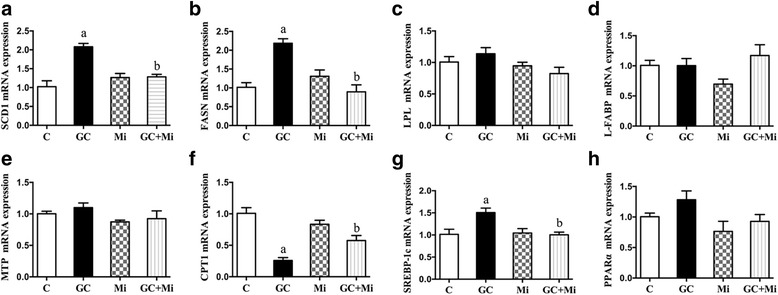
mRNA expression of SCD1 (**a**), FASN (**b**), LPL (**c**), L-FABP (**d**), MTP (**e**), CPT1 (**f**), SREBP-1c (**g**) and PPARα (**h**) in GC or Mi or GC + Mi treatment. Data are expressed as the mean ± SEM. Effects of different treatments were analyzed by two-sided Student’s *t*-test. ^a^*p* < 0.05 vs. control, ^b^*p* < 0.05 vs. GC. *n* = 3 in each treatment

**Fig. 7:**
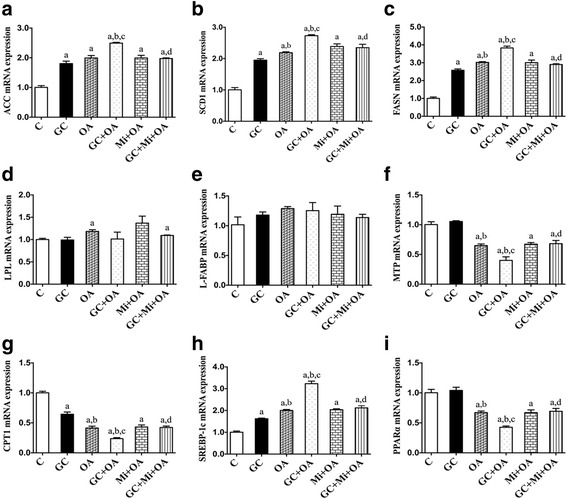
mRNA expression of ACC (**a**), SCD1 (**b**), FASN (**c**), LPL (**d**), L-FABP (**e**), MTP (**f**), CPT1 (**g**), SREBP-1c (**h**) and PPARα (**i**) in GC or OA or GC + OA or Mi + OA or GC + Mi + OA treatments. Data are expressed as the mean ± SEM. Effects of different treatments were analyzed by two-sided Student’s *t*-test. ^a^*p* < 0.05 vs. control, ^b^*p* < 0.05 vs. GC, ^c^*p* < 0.05 vs. OA, ^d^*p* < 0.05 vs. GC + OA. *n* = 3 in each treatment

To determine whether the regulation of hepatic lipid accumulation by GC was mediated by metabolism enzymes, we next examined ACC, SCD1, FASN and SREBP-1c mRNA expression in the HepG2 cells. As expected, the level of ACC mRNA was dependent on the dose and timing of the GC stimulation (*p* < 0.05, Fig. 5a, b); the optimum concentration and timing were 125 nM for 48 h. SCD1, FASN and SREBP-1c mRNA expression increased in GC stimulation (*p* < 0.05, Figs. 6a, b, g), but CPT1 expression decreased (*p* < 0.05, Fig. 6f), and all these alterations were more significant in the GC + OA treatment (*p* < 0.05, Fig. 7b, c, g, h), as well as ACC (*p* < 0.05, Fig. 7a). Mi alone (*p* > 0.05, Fig. 6) and the Mi + OA treatment (*p* > 0.05, Fig. 7) did not have any effect on the expression of these genes, but Mi attenuated GC or GC + OA induced up-regulation of ACC, SCD1, FASN and SREBP-1c expression (*p* < 0.05, Figs. 5c, 6, 7) and down-regulation of CPT1 expression (*p* < 0.05, Figs. 6, 7).

## Discussion

It has become increasingly recognized that the metabolic programming effects of nutritional modifications in early postnatal life are independently related to the development of obesity and MS in later life [31]. Over-nutrition during lactation induces a persistent increase in body weight, hyperinsulinemia, hyperleptinemia and MS in adults, including NAFLD [30, 31]. Consistent with our previous reports, we confirmed that early neonatal overfeeding induced increased mRNA expression of 11β-HSD1, decreased expression of 5αR1 and 5βR and abnormal lipid metabolism in the livers of the SL rats compared to NL rats. The new finding of this study was that exposure to GC increased hepatocyte lipid accumulation by up-regulating the gene mRNA expression of hepatic DNL through GR. We suggest that early postnatal overfeeding induced by SL rearing leads to peripheral GC metabolism activity, which might contribute to the increase in hepatic lipid synthesis in adult rats.

Previous studies have shown the presence of higher 11β-HSD1 mRNA and/or activity in the adipose tissue of obese rodents [24, 25] or humans [23, 26]. 11β-HSD1 is known to be positively associated with features of MS in adults [20]. Transgenic mice with adipose- or liver-specific 11β-HSD1 overexpression exhibit elevated intra-adipose and portal corticosterone levels, abdominal obesity, dyslipidaemia, insulin resistance and hypertension [44, 45]. In our studies, SL adult rats exhibited obesity and increased hepatic 11β-HSD1 overexpression but decreased 5αR1 and 5βR expression, indicating that there are more active GC in hepatic tissue. The high concentrations of GC in the liver could have important effects on lipid metabolism [28, 45, 46]. In the present study, small litters displayed significant increases in liver mass and TG contents compared to NL rats.

GC can lead to hepatic steatosis by decreasing lipid export and oxidation [47, 48], increasing cholesterol synthesis and fatty acid uptake [49, 50] or increasing lipid biosynthesis [28, 29]. In addition to the changes of 11β-HSD1, 5αR1 and 5βR in the liver, we also found that postnatal overfeeding induced a significant increase in DNL by SREBP-1c, ACC, SCD1 and FASN overexpression in the liver from weaning to adulthood, which might be an important mechanism underlying the development and progression of NAFLD in adulthood, that is, overexposure to GC through 11β-HSD1 up-regulation in the liver.

In line with our hypothesis that overexposure to GC induces an increase in DNL in hepatocytes, we found that both lipid accumulation and TG content in HepG2 cells were significantly increased by GC treatment via DNL increase through SREBP-1c, ACC, SCD1 and FASN overexpression. Therefore, the augmented active GC induced by the increase in 11β-HSD1 might be an important factor responsible for the increased DNL in the livers of SL-reared rats. Because the effects of GC were mainly mediated via the GR, which is a member of the steroid hormone receptor superfamily [51, 52], we used Mi, the GR antagonist [53, 54], and confirmed that most of the hepatic lipid metabolism changes induced by GC were inhibited by Mi. Thus, we suggest that GC could increase lipid accumulation by increasing DNL through its receptor in the hepatocytes.

Although postnatal overfeeding can alter lipid metabolism in the liver, a high-fat diet is central to the onset of NAFLD [55, 56]. In our previous studies, we found that neonatal overfeeding in rats induced by SL rearing increased their vulnerability to a HF diet from post-suckling to adulthood and promoted early onset and exaggeration of HF diet-induced NAFLD [38]. Moreover, we found that SL and a high-fat diet exhibited a significant interaction with regard to 11β-HSD1 expression, but hepatic 11β-HSD1 expression was not observed in NL-HF rats [57]. We suggest that the the increased activity of the GC induced by 11β-HSD1 and a HF diet have a significant interaction on lipid metabolism in the liver. In the present study, we found that the GC + OA treatment in vitro resulted in the most significant lipid accumulation and DNL increase in HepG2 cells compared to separate GC or OA treatments.

Previous studies have shown that during energy overconsumption, LPL and L-FABP expression increased in the liver [58, 59], but CPT1and MTP decreased [60, 61]; all these alterations could contribute to the occurrence of NAFLD [27, 62]. In the present study, we found that CPT1 decreased after GC treatment in vitro, but it did not change at W13 in the SL rats. Notably, our previous observation indicated that CPT1 decreased at W16 in the SL rats [38], suggesting that long-term overexposure to GC also affected lipid oxidation in the hepatocytes. Furthermore, the transient elevation of LPL and L-FABP mRNA expression in the SL rats (at W3) might be due to the excessive food intake; it did not change after weaning in vivo or after GC overexposure in vitro. There was also no change in MTP or PPARα caused by GC overexposure either in vivo or in vitro. Therefore, we suggest that GC overexposure in the SL rat model and HepG2 cells augmented the hepatic lipid accumulation mainly through DNL increase.

## Conclusions

Postnatal overfeeding induced GC overexposure through 11β-HSD1 up-regulation in the liver, and the GC activated the hepatic DNL by GR. This resulted in hepatic lipid accumulation, leading to an increased risk of NAFLD during adulthood. More animal and clinical studies are needed to examine the prolonged effects of manipulating the availability of pre-receptor GC and the mechanisms of GR activation in the liver. Specifically, we suggest that targeting pre-receptor GC activation in the liver may provide a novel approach to the treatment of NAFLD, particularly in childhood.

## Abbreviations

11β-HSD1: 11β-hydroxysteroid dehydrogenase 1
5αR1: 5α-reductase type 1
5βR: 5β-reductase
ACC: Acetyl-CoA carboxylase 1
ANOVA: Analysis of variance
CPT1: Carnitine palmitoyltransferase
DNL: De novo lipogenesis
FASN: Fatty acid synthase
GAPDH: Glyceraldehyde-3-phosphate dehydrogenase
GC: Glucocorticoid
GR: Glucocorticoid receptor
HF: High-fat
HSI: Hepatosomatic index
L-FABP: Liver fatty acid-binding protein
LPL: Lipoprotein lipase
Mi: Mifepristone
MS: Metabolic syndrome
MTP: Microsomal triglyceride transfer protein
NAFLD: Non-alcoholic fatty liver disease
NL: Normal litter
OA: Sodium oleate
P3: Postnatal day 3
PPARα: Peroxisome proliferator-activated receptor α
SCD1: Stearoyl-CoA desaturase-1
SL: Small litter
SREBP-1c: Sterol regulatory element binding protein-1c
TG: Triglyceride
W13: Postnatal week 13
W3: Postnatal week 3
